# Powering up antifungal treatment: using small molecules to unlock the potential of existing therapies

**DOI:** 10.1128/mbio.01073-23

**Published:** 2023-08-02

**Authors:** Rebecca S. Shapiro, Aleeza C. Gerstein

**Affiliations:** 1 Department of Molecular and Cellular Biology, University of Guelph, Guelph, Ontario, Canada; 2 Department of Microbiology, University of Manitoba, Winnipeg, Manitoba, Canada; 3 Department of Statistics, University of Manitoba, Winnipeg, Manitoba, Canada; Duke University, Durham, North Carolina, USA

**Keywords:** antifungal therapy, antifungal resistance, mycology, antimicrobial combinations, drug screening

## Abstract

Fungal pathogens are increasingly appreciated as a significant infectious disease challenge. Compared to bacteria, fungal cells are more closely related to human cells, and few classes of antifungal drugs are available. Combination therapy offers a potential solution to reduce the likelihood of resistance acquisition and extend the lifespan of existing antifungals. There has been recent interest in combining first-line drugs with small-molecule adjuvants. In a recent article, Alabi et al. identified 1,4-benzodiazepines as promising molecules to enhance azole activity in pathogenic *Candida* spp. (P. E. Alabi, C. Gautier, T. P. Murphy, X. Gu, M. Lepas, V. Aimanianda, J. K. Sello, I. V. Ene, 2023, mBio https://doi.org/10.1128/mbio.00479-23). These molecules have no antifungal activity on their own but exhibited significant potentiation of fluconazole in azole-susceptible and -resistant isolates. Additionally, the 1,4-benzodiazepines increased the fungicidal activity of azoles that are typically fungistatic to *Candida* spp., inhibited filamentation (a virulence-associated trait), and accordingly increased host survival in *Galleria mellonella*. This research thus provides another encouraging step on the critical pathway toward reducing mortality due to antimicrobial resistance.

## COMMENTARY

Fungal pathogens pose an immense and often underappreciated threat to human health (1), with 150 million life-threatening fungal infections and 1.5 million annual deaths from fungal disease (2, 3). The WHO recently released the first *Fungal Priority Pathogens List*, an acknowledgment of the immense global challenge that fungi pose (4) and highlighting four critical fungal pathogens that cause life-threatening invasive mycoses: *Candida albicans*, *Candida auris*, *Cryptococcus neoformans*, and *Aspergillus fumigatus*. Invasive fungal infections pose a significant threat, particularly among people with chronic medical conditions or immunodeficiencies, including cancer and transplant patients, those living with HIV/AIDS, and diabetics. As the population of vulnerable individuals is increasing, so too has the incidence of fungal infections (5). This became uniquely salient during the COVID-19 pandemic, where large populations of high-risk, hospitalized individuals, along with corticosteroid use, enabled fungal diseases to proliferate rapidly. This caused a rapid increase in disease burden globally (6), including a devastating mucormycosis outbreak in India (7).

Antifungal treatments for invasive fungal infections are primarily limited to three major classes of drugs: polyenes, azoles, and echinocandins (8). These limited antifungals suffer from important concerns including patient side effects, drug interactions, limited spectrum of action, and poor bioavailability. One major hurdle associated with antifungal drug development is the close evolutionary relationship between eukaryotic fungi and their human hosts, limiting our ability to identify clinically effective, non-toxic therapeutics. Indeed, polyenes, the oldest class of drugs, have exhibited relatively low levels of resistance emergence (9, 10), but are associated with severe side effects, including renal toxicity (11). Azoles are the largest drug class and are currently the most commonly administered. They have a broad spectrum of activity and can be orally administered with fewer side effects. Unfortunately, azole resistance seems to be increasing over time among diverse species (12, 13), and isolates of many of the non-*C*. *albicans* species have intrinsically high levels of azole resistance, including *Nakaseomyces glabrata* (formerly *Candida glabrata*), *Pichia kudriavzevii* (formerly *Candida krusei*), and *C. auris* (14, 15). Furthermore, azole drugs are fungistatic against many important pathogens and primarily inhibit cell growth rather than kill susceptible cells (8, 16). Antifungal drug tolerance, the ability of a subpopulation to grow slowly under fungistatic drug pressure, is a distinct drug response phenotype from drug resistance, and it has recently been proposed as having potential involvement in recalcitrant infections from drug-susceptible isolates that are not cleared following standard courses of treatment (17). The newest class of drugs, the echinocandins, is very effective against the majority of important human fungal pathogens (except *C*. *neoformans* and *Fusarium* spp. (18, 19)). They unfortunately suffer from poor oral bioavailability, and must be administered intravenously (20), which limits their use in some resource-poor settings (21). Combined, these limitations underscore the urgent need to develop new therapies with novel mechanisms of action or to use alternative treatment approaches to extend the utility of existing drugs.

Although there are finally some new drugs in the antifungal pipeline (22), the identification and development of new antimicrobial drugs is a long and difficult process. There has thus been a strong push to evaluate methods to maximize the use of existing therapeutics that have already been approved. Combination therapy pairs multiple drugs with similar pharmacokinetics, unpaired toxicity, and minimal potential for cross-resistance (23). This strategy has been used to successfully reduce mortality from many diverse illnesses such as HIV and malaria (23) and Gram-negative bacteria (24). Combination therapy offers potential improvements over monotherapy in a number of important regards, including synergistic interactions, a reduction of toxicity (due to lower dosing of individual drugs), a shorter duration of treatment, and the potential for rejuvenation or reuse of antibiotics that are no longer employed due to high rates of resistance (25). Combination therapy from the front-line antifungals is rarely employed clinically; however, excepting the use of amphotericin B and flucytosine to treat cryptococcal meningitis (26). Antifungal drug combinations have been limited due to the potential for antagonistic behavior and increased toxicity, and *in vitro* results have not always been recapitulated *in vivo*. Ultimately, it is difficult and expensive to conduct clinical trials for antifungals, limiting the availability of clinical results to inform combination therapy in practice (27 - 29).

Nevertheless, as a promising approach to overcome the limitations of the existing antifungal monotherapies, researchers have recently turned to exploring combination therapy with small molecules and adjuvants. In broad terms, an adjuvant is any substance (or combination thereof) that can increase the potency or efficacy of a drug. One promising class of adjuvants is small molecules, low molecular weight organic compounds that are small enough to diffuse across cell membranes. Small molecules can work to enhance the host immune response, or to enhance the efficacy of existing antifungal drugs in multiple ways: they can increase drug availability in the cell through increasing drug entry or decreasing degradation; they can target the same biological pathway, typically at different stages; or, they can target different proteins in parallel pathways that influence the same cellular process (30). Multiple approaches have been developed to efficiently screen small molecule collections for compounds that exhibit synergistic behavior with antifungal drugs (most commonly azoles), yielding promising targets and results (31 - 34).

In their recent study, Alabi et al. (35) identified a novel set of molecules with synergistic behavior to azoles for combination antifungal therapy. They performed a small molecule screen that identified novel 1,4-benzodiazepines that demonstrated no antifungal activity on their own, but that were able to uniquely potentiate the activity of azole antifungals. These powerful potentiators significantly enhanced fluconazole activity, and rendered the fungistatic azoles fungicidal against *C. albicans* and other closely related *Candida* pathogens. Critically, the synergistic activity of the 1,4-benzodiazepines and azoles was demonstrated in azole-susceptible, -resistant, and -tolerant *C. albicans* isolates, suggesting an important application of these drugs in treating otherwise recalcitrant infections.

While the precise mechanism by which the 1,4-benzodiazepines potentiate azole activity remains to be fully elucidated, evidence from Alabi et al. suggests a role for lipid homeostasis, as cells treated with both compounds displayed an increase in lipid droplets in the fungal cell. Lipid droplets are known to play an important role in the cellular response to stress, including nutrient, oxidative, ER, and others (36). It is known that stress responses are vital for fungal cells to tolerate antifungal drug treatment. The connection between 1,4-benzodiazepines and the fungal stress response is reminiscent of other compounds that have synergistic activity with antifungal agents and target stress response pathways (37). Indeed, pharmacological inhibitors of key stress response factors, including the molecular chaperone Hsp90 and the protein kinase calcineurin, have previously been shown to have promising activity as potentiators of antifungal activity against *Candida* pathogens (38, 39).

In addition to their synergistic activity with azoles, Alabi et al. also identified a role for the 1,4-benzodiazepines in impairing the ability of *C. albicans* to transition from yeast to filamentous growth morphologies. This transition is critical for *C. albicans* virulence, as filamentous cells express numerous virulence factors (40), and strains that are unable to transition between states have reduced virulence in animal models of infection (41). Alabi et al., used *Galleria mellonella* moth larvae as a tractable model of *C. albicans* infection and found that treatment with 1,4-benzodiazepines provided a modest improvement in survival for fungal-infected larvae. There is burgeoning interest in the development of such “antivirulence” compounds as therapies for fungal infections, and the yeast-to-filamentous growth transition in *C. albicans* is a prime target for such therapeutics (42). It is suggested that compounds targeting microbial virulence should exert a weaker selection pressure than conventional antimicrobials that impair viability, resulting in decreased frequency of resistance. Further, studies in bacterial pathogens suggest that antivirulence compounds can act synergistically with anti-bacterial agents and potentiate the activity of these antibiotics amongst drug-resistant strains (43). This is consistent with the phenotypes observed by Alabi et al., which suggest that targeting virulence may be a similarly powerful strategy to not only reduce fungal pathogenicity but to enhance the activity of existing antifungal agents.

This study builds upon a growing body of literature highlighting the need to identify novel compounds to potentiate existing antifungal drugs. In addition to chemical screening approaches such as those employed by Alabi et al., other strategies may further enhance our understanding of how to optimally deploy antifungal treatments. Chemical-genetic and genetic interaction screens are highly complementary to drug screening and can identify new fungal pathways to target for combination therapeutics (44, 45). Compelling research in bacterial and fungal species has exploited genetic interaction studies to identify combinations of mutations that synergize to impair microbial fitness (45 - 47), and identify mutations that sensitize drug-resistant strains to antimicrobial agents (45, 48 - 50). These strategies can ultimately be translated to predict novel combinations of drugs that target the identified genes or pathways, which may act as therapies against microbial infections. Leveraging the pleiotropic effects associated with resistance is another innovative way to uncover combination therapies against drug-resistant pathogens. Research in bacterial species has found that the evolution of resistance to specific antibiotics renders cells more highly susceptible to other classes of antibiotics, and proposes that we can leverage this “collateral sensitivity” to better exploit combinations of existing antimicrobials to treat resistant infections (51, 52).

With the challenges associated with new antifungal drug development and increasing rates of clinical antifungal resistance, it is clear that to combat antifungal resistance we need to maximize the use of existing approved antifungals. Combination therapy with small molecules such as the 1,4-benzodiazepines identified by Alabi et al. represents a promising avenue for much-needed novel treatment strategies.
